# Solitary Osteochondroma at Unusual Sites: A Case Report and Literature Review

**DOI:** 10.7759/cureus.49582

**Published:** 2023-11-28

**Authors:** Faisal A Alghamdi, Nibras K Aljabri, Hasan M Jafar, Abdulkhaleq H Almatari, Salem A Bajuifer

**Affiliations:** 1 College of Medicine, Umm Al-Qura University, Makkah, SAU; 2 Orthopedic Surgery, Al-Noor Specialist Hospital, Makkah, SAU

**Keywords:** tumor, malignant transformation, scapular osteochondroma, exostosis, case report, bone neoplasm, scapula, osteochondroma

## Abstract

Osteochondromas (OCs) are bone lesions composed of cartilaginous and medullary bone capped with hyaline cartilage. OCs result from the separation of epiphyseal growth plate cartilage, pushing through the periosteal bone cuff. They commonly appear as pedunculated or sessile masses in the metaphysis of long bones and are the most common benign bone tumors. While rare in the scapula, OCs can occur there. Symptoms may arise from fractures, osseous abnormalities, or potential malignant transformation, especially in the presence of hereditary multiple exostoses (HME). The estimated rate of malignant transformation in solitary lesions is 1%, whereas in hereditary multiple OCs, it can reach up to 3-5%.

We report a case of a 10-year-old female who presented with a gradually progressive swelling on the back of her right scapula. This progressive growth has been observed over the course of the past two years accompanied by mild pain. The pain was intermittent and did not affect her daily activities. On examination, a hard, tender, non-mobile swelling of approximately 2 × 2 cm was found over the right scapula. The patient had a normal range of motion in the shoulder and scapulothoracic regions.

In conclusion, since solitary scapular OCs are extremely rare, they are quite common when associated with HME. This study aimed to increase awareness of the unusual site of OCs. Furthermore, we have included a full account of the surgical therapy we administered to this patient in order to assist future surgeons who may come across similar conditions.

## Introduction

Osteochondromas (OCs) are bone lesions comprising cartilaginous and medullary bone capped with hyaline cartilage. The presence of the medullary cavity of the lesion is continuous with the medullary cavity of the native bone, which is considered pathognomonic for OCs [1-4]. Instead of being real neoplasms, OCs are more like exostotic, developing lesions. OCs form when a piece of the epiphyseal growth plate cartilage separates and pushes through the periosteal bone cuff around the growth plate [2,5]. Previous studies on OCs have reported a prevalence rate ranging from 1% to 3%. These estimates were derived from assessments of patients who underwent thorough radiographic evaluations and from pathology series [6]. However, it should be noted that there are no precise statistics or prevalence data available regarding the extent of OCs spread in the Kingdom of Saudi Arabia (KSA). Multiple differential diagnoses can be considered, such as Nora’s lesion, Dupuytren’s exostosis, turret exostosis, and subperiosteal hematoma; none of these have medullary continuity [1-4,7-11]. Furthermore, OCs are the most common primary bone tumors, comprising approximately 40% of all benign tumors. Some common sites for OCs are the proximal humerus, proximal tibia, and distal femur [12,13]. OCs can manifest as either a pedunculated or sessile mass, with the pedunculated form being more prevalent [14]. Usually mushroom-shaped, the lesion affects the metaphysis of long bones, such as the femur and tibia [15]. Although OCs are a rare and intriguing occurrence in the scapula, comprising approximately 4.6% of all bone tumors [16,17]. Symptoms can also be caused by osseous abnormalities, fractures, bursa formation, mechanical compression of nearby structures, or even malignant transformation [3,4,18,19]. Malignant transformation has an infrequent but notable association with hereditary multiple exostoses (HME) [18]. This condition predominantly affects the long bones in the leg - femur, humerus, and pelvis.

We discuss the case of a 10-year-old female with a mass in the dorsal scapular OC, which is rare for this age and gender. Little information is available on dorsal scapular OC. However, excision was chosen in every publicly disclosed case and has proven effective in resolving symptoms [12,17,20,21].

## Case presentation

**Figure 1:**
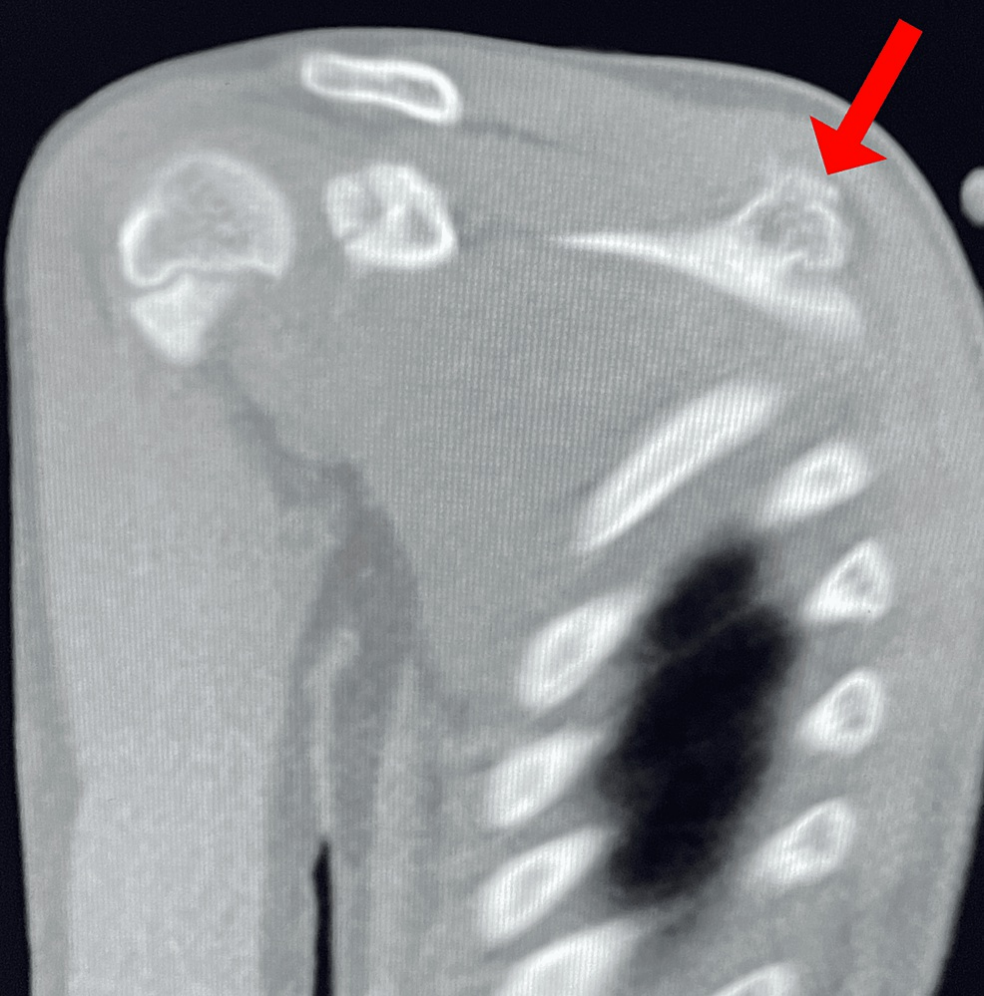
CT scan showing abnormal bone growth. The image shows an exostosis of the right scapula (arrow).

**Figure 2:**
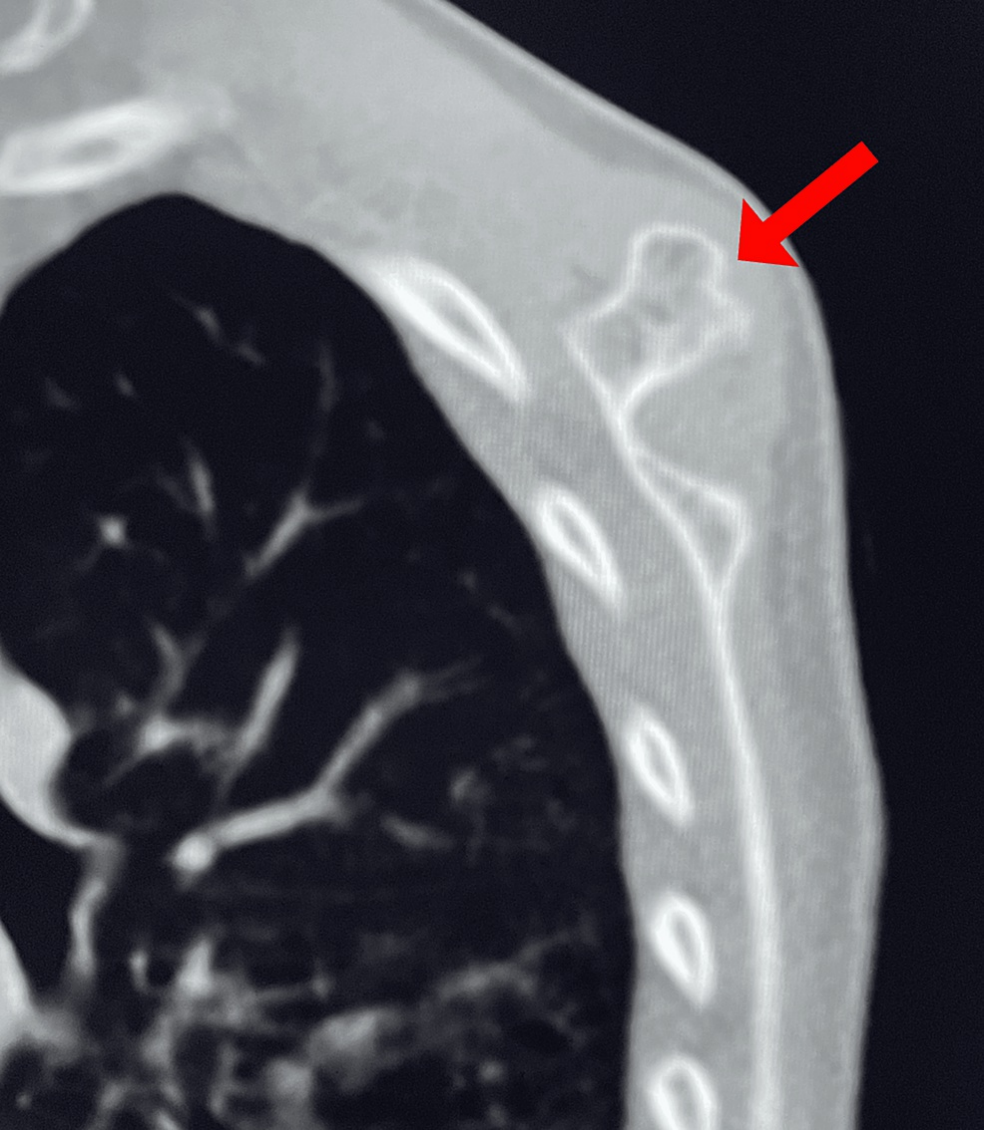
CT scan reveals abnormal bone growth. The image shows a posterior exostosis of the right scapula (arrow).

A healthy 10-year-old female patient not known to have any medical illnesses presented in our outpatient department with complaints of swelling at the back of the right scapula for two years. The swelling was insidious at onset, gradually progressive, and associated with slight pain around the lump. The pain was dull and not aggravating, and the patient did not take any painkillers and received no radiation. The pain was intermittent, did not worsen over time, and did not affect the patient’s daily activities. On examination, there was a hard swelling of approximately 2 × 2 cm over the right back of the scapula at the medial upper border. The swelling was tender, non-mobile, and fixed to the underlying bone but not adherent to the skin or overlying tissue. It was not associated with redness or hotness. The patient had a full range of motion in the shoulder and scapulothoracic regions without any accompanying snapping sensation. There was no family history of the illness except for her sister having a breast tumor. She lived with her parents, who were financially stable. To exclude other diagnoses, the patient was referred for a full investigation, including an X-radiography (XR), computed tomography (CT), and magnetic resonance imaging (MRI). A CT scan was performed on the patient and showed evidence of a bony outgrowth on the superior medial edge of the right scapula (Figure 1). It had a posteriorly oriented bony outgrowth lesion and a continuous cortex and medullary cavity with the parent bone (Figure 2). The right shoulder joint articulation was present.

**Figure 3:**
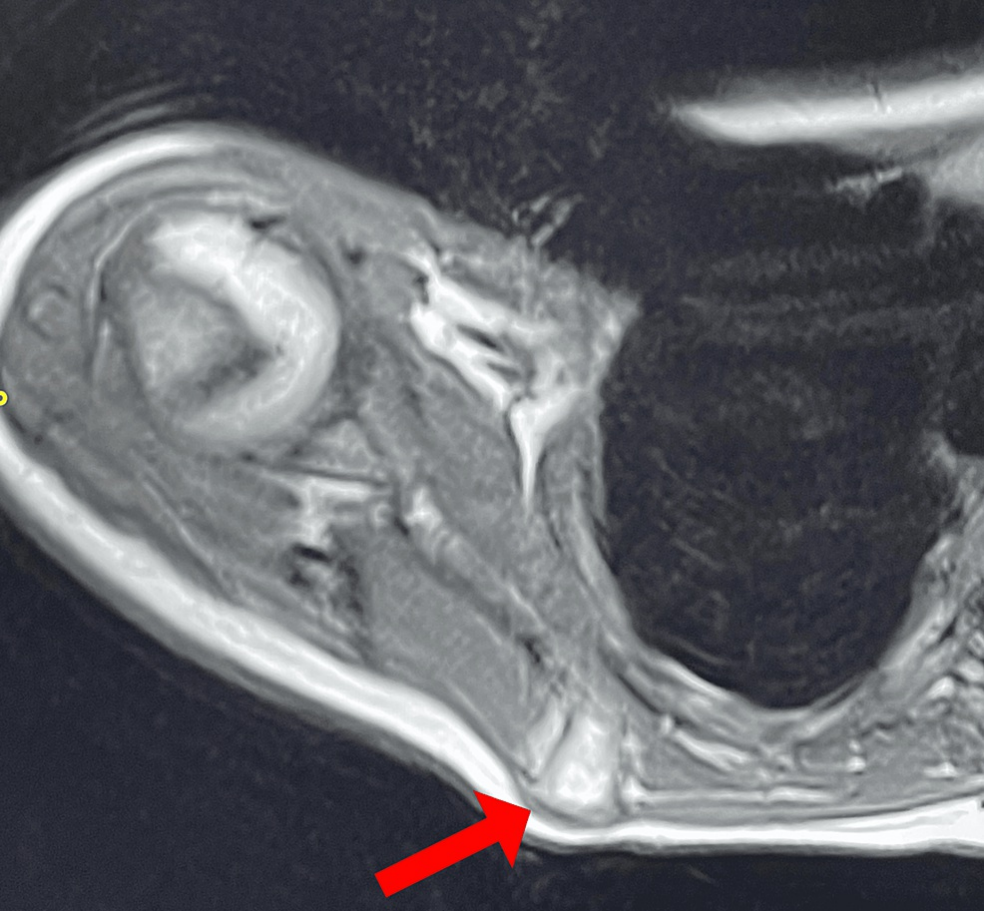
MRI showing abnormal bone growth. The image shows an exostosis of the right scapula (arrow).

**Figure 4:**
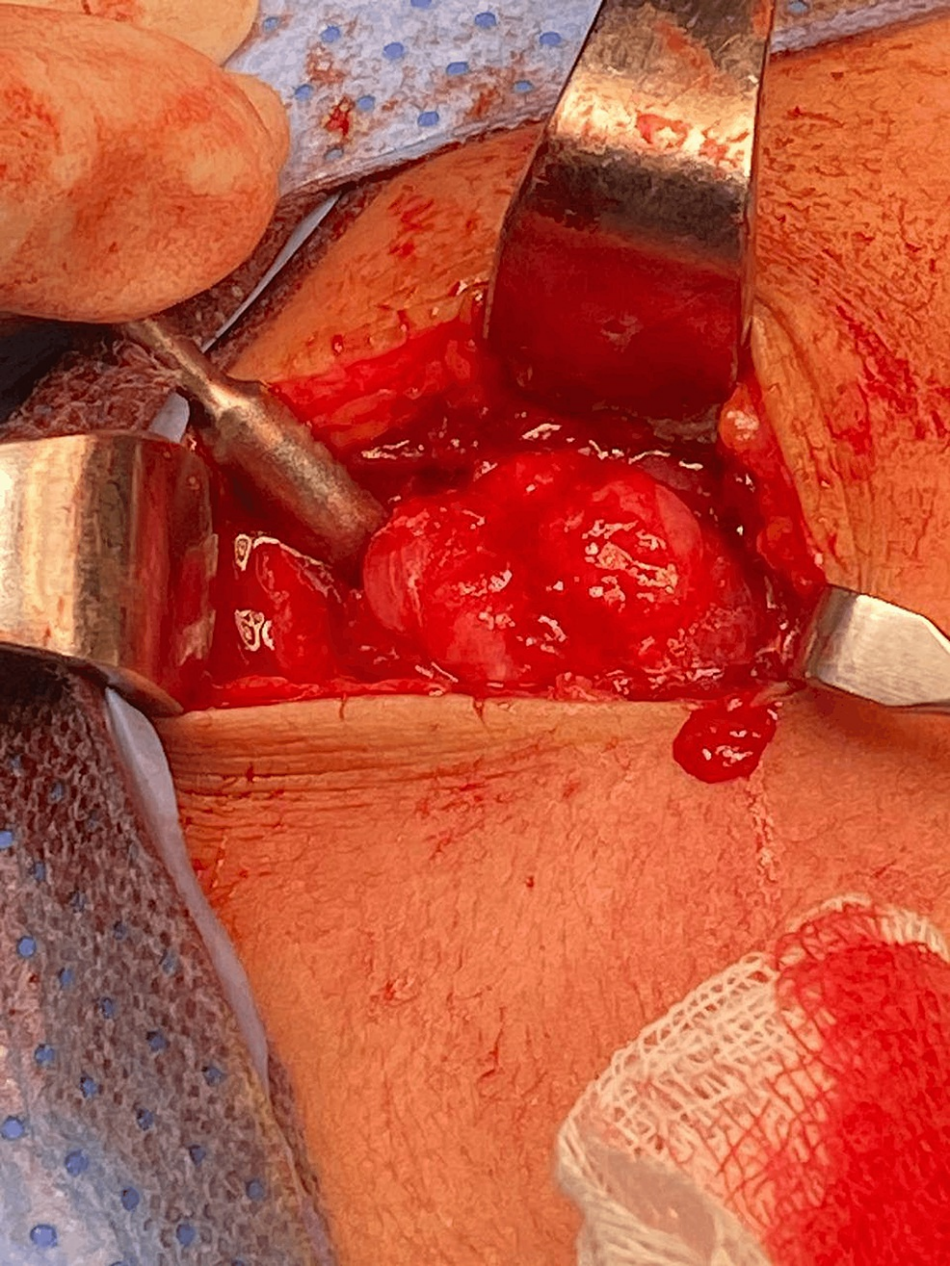
Surgical removal of the mass.

The MRI showed bony exostosis with cortical and medullary continuation, most likely OCs, measuring 1.6 cm craniocaudal (CC), 1.5 cm AP, and 1.6 cm transfers, with a cartilage cap of 0.3 cm (Figure 3). It was determined that surgical resection with adequate margins would be undertaken, primarily for both cosmetic indications and to minimize the potential for malignant transformation. The patient was admitted to the day surgery department and placed under general anesthesia. Preparation and draping over the surgical site were undertaken. Then, an oblique incision was made around 4-6 cm superior to the scapula spine using a direct approach (Figure 4). Continuing to cut through the subcutaneous tissue exposed the supraspinatus muscle.

**Figure 5:**
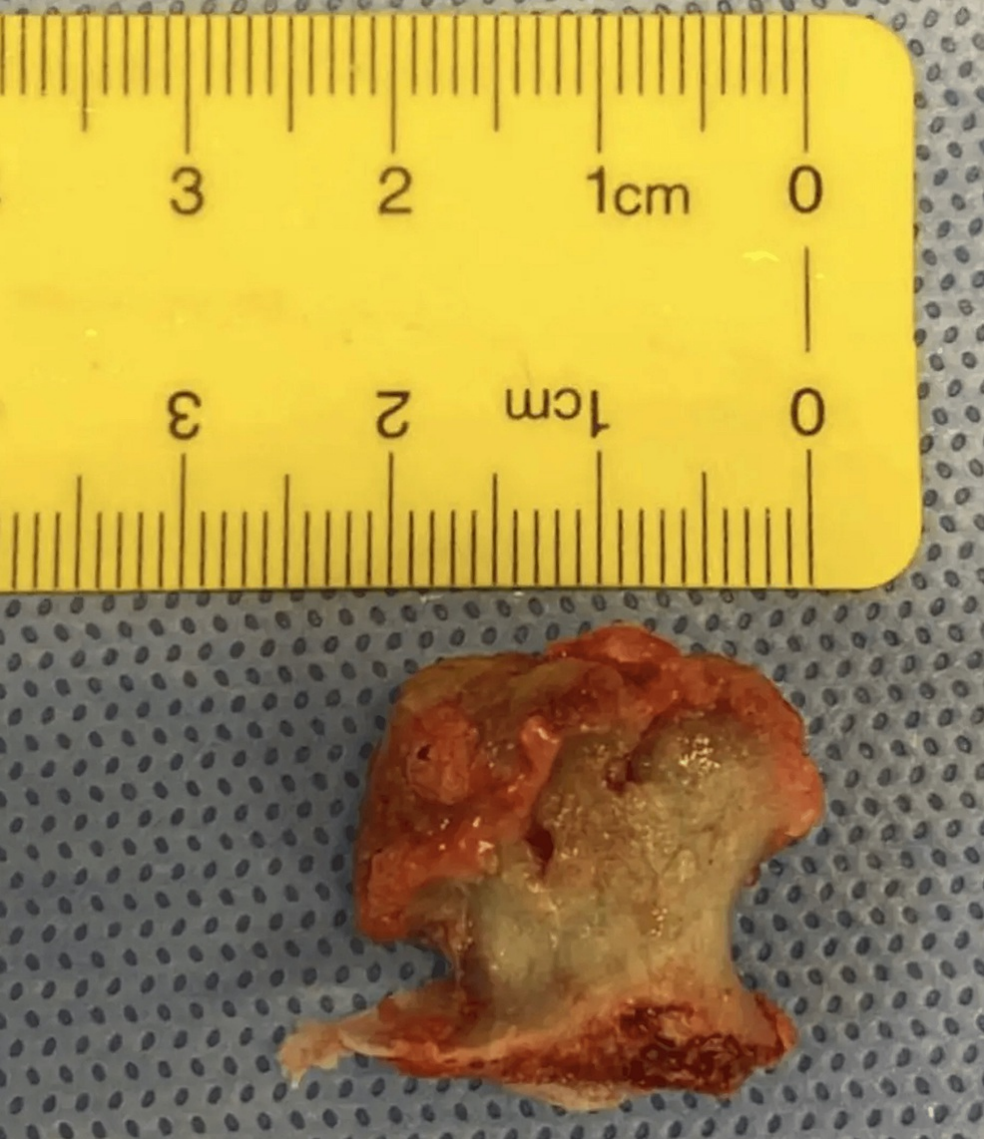
The exostosis is surgically removed from its base with an appropriate safety margin.

After removal, the bony mass was measured approximately 2 cm in size. It was covered with a cartilage cap arising through the muscle fibers from a base in the supraspinatus fossa. All muscle adhesions and attachments to the lesion were released, and the mass was then excised (Figure 5). The specimen measured 2 × 2 cm, and its cartilage cap was 2.2 cm. The mass was then sent to the histopathology lab in a formalin solution. A wash and closure were undertaken after the approximation of muscle fibers. Sutures were performed by inverting vicryl 4-0 and closing the skin with polypropylene 4-0. The patient was then transferred to the recovery room.

The mass was sent for histopathology, which indicated that the cartilage cap had not undergone any malignant changes. An OCs diagnosis was supported by a microscopic examination, which showed foci of endochondral ossification in sections of normal bone tissue. The postoperative course for the patient was uneventful, and the patient made a full recovery without any lingering discomfort, edema, or scapular winging. She began rehabilitation therapy after her surgery and continued it as an outpatient. At a follow-up one year after surgery, radiographs revealed that she was still doing well, with complete shoulder mobility, no reported recurrence of symptoms, and no scapular winging.

## Discussion

As previously mentioned, OCs of the scapula represent 4% of all bone tumors [15,16]. Since OCs do not often cause symptoms, and their development ceases when the physis closes, they are typically discovered in the first or second decades of life [21,22]. This case involves an asymptomatic mass that the mother of a 10-year-old child noticed. This mass was comparable to those reported in several other studies in the literature, considering other age groups, including having a similar presentation [11,16,23-30]. OCs of the anterior surface of the scapula present as a winging prominent scapula, commonly seen in HME, where surgical management is demanding. Our case is unique because it involves a female with OC, which is uncommon in the literature (Table 1).

**Table 1:** Literature review showing similar cases. XR: X-radiography

Year	Complaint	Gender	Age (years)	Location	Diagnoses modality	Size	Histopathology	Operative procedure
Our case 2023	Cosmetic mass	F	10	Right	MRI + CT	2 × 2 cm	Hyaline cartilage cap overlaying the stalk	Excisional biopsy
2019 [11]	Painless mass	M	2	Right	XR	8.4 × 7.2 cm	Confirmed osteochondroma of the scapula	Complete resection of osteochondroma
2014 [16]	Progressive swelling and movement restriction	M	4	Left	XR	30 × 17 mm	Confirmed osteochondroma of the scapula	Complete resection of osteochondroma
2022 [18]	Painful mass over left scapula	F	2	Left	CT + XR	3 × 2.5 cm	Confirmed osteochondroma of the scapula	Excised the mass
2014 [19]	Large, painless deformity, scapular winging, limited abduction	M	18	Right	CT	5 × 3 cm	Well-formed cartilage cap on the surface with a prominent endochondral ossification at the base, which continues into the trabeculae of mature lamellar bone	Excised specimen showing a bony tumor with a cartilaginous cap
2019 [20]	Worsening shoulder pain with right scapula winging	M	17	Right	CT	9 × 5 cm	Confirmed osteochondroma of the scapula	The stalk of the exostosis was excised
2021 [23]	Painful mass	M	11	Right	MRI	4 × 2.8 cm	Confirmed osteochondroma of the scapula	After partially detaching the teres minor muscle, the tumor mass was accessed and resected
2019 [24]	A mass on the upper back, inability to sleep in a supine position, painful range of shoulder motion, and cosmetic discomfort	F	15	Left	CT + XR	N/A	Confirmed osteochondroma of the scapula	Mass was excised using osteotome
2016 [26]	Difficulty sleeping in the supine position	M	12	Right	XR + CT	4 × 3 cm	Confirmed osteochondroma of the scapula	Excised the mass
2021 [27]	Painless long-standing protrusion over the scapula	M	23	Left	MRI	N/A	N/A	No surgery done yet
2018 [28]	Progressive swelling and discomfort in the supine position	M	19	Left	CT + XR	3 × 3 cm	Confirmed osteochondroma of the scapula	Complete resection of osteochondroma
2018 [28]	Progressive swelling	M	5	Left	XR	1.5 × 1 cm	Confirmed osteochondroma of the scapula	Complete resection of osteochondroma
2018 [29]	Shoulder discomfort	M	25	Right	CT + MRI	3 × 2 cm	Confirmed osteochondroma of the scapula	Complete resection of osteochondroma
2007 [30]	Cosmetic mass	M	13	Left	CT + XR	4 × 4 cm	Confirmed osteochondroma of the scapula	Excised the mass

Some studies have reported worsening pain [20-23]. Others have reported having trouble falling asleep in a supine posture [22-23], and some studies have shown restrictions in the range of motion of patients’ joints [16-19]. Larger lesions often occur on the inferior part of the scapula because of a lack of space. Most osteochondral lesions of the scapula are located along the scapular equator [30,31]. Histological confirmation usually follows a clinical and radiographic diagnosis of OC [30,32].

New cortical irregularities, growth continuing after skeletal maturity, bony destruction, the back growth of the cartilaginous cap into the stalk or medullary canal, the lysis of calcifications in the cap, focal regions of radiolucency inside the lesion, and a large soft tissue mass are radiographic indications of malignant transformation. Lesions of 1-2 cm are suspicious [17], and an increased cartilage cap thickness exceeding 3 cm in children or 2 cm in adults is a sign of malignant transformation [33]. However, understanding that skeletal maturity significantly influences cartilage cap size is crucial. Larger caps are characteristic of skeletal immaturity and also indicate active growth and malignant transformation [21].

The presence of OCs should not be misconstrued as a sign of cancerous transformation [17]. However, clinical signs such as pain, swelling, irregular borders, and growth of the mass may indicate malignant transformation, particularly after a period of dormancy. It is important to note that these indications primarily apply to young adults rather than children. Since the ilium, scapula, and pubic rami are the sites most often linked to malignant alteration, OCs in these areas should raise concerns about their potential for malignancy [17]. OCs do not metastasize; the estimated rate of malignant transformation in solitary lesions is 1%, whereas in HME, it can reach up to 3-5% [1].

Given that longitudinal growth causes a tumor to migrate from the metaphysis to the diaphysis and away from the growth plate, decreasing the likelihood of injuring the growth plate, OCs are typically managed after skeletal maturity [18,34]. If a patient’s primary concerns with scapular exostosis are pain and cosmesis, as they were in our case, excision can be performed at a younger age, provided it is well planned and conducted by an experienced surgeon.

## Conclusions

Scapular osteochondromas (OCs) are non-cancerous tumors that can occasionally transform into malignant forms. To avoid possible complications like malignant transformation, especially when there is an increase in cartilage cap, it is advisable to consider surgical removal of scapular OCs, whether to relieve any existing symptoms or to reduce the chance of malignant transformation. Following a diagnosis, the excision procedure should be conducted with great care, aiming for complete removal at the base of the stalk while minimizing the risk of harm to nearby muscles and vital structures. Fortunately, in the case described, the patient experienced a successful recovery, and the overall outcome was positive. This highlights the importance of timely intervention and meticulous surgical techniques in managing scapular OCs, ensuring optimal outcomes for patients.
